# ATF3/SLC31A1-Mediated Cuproptosis Contributes to Bortezomib-Induced Peripheral Neurotoxicity and Intervention by (−)-Epigallocatechin Gallate

**DOI:** 10.3390/ijms27083680

**Published:** 2026-04-21

**Authors:** Yonghai Wang, Jiabin Lu, Xuejing Feng, Bo Yang, Qiaojun He, Peihua Luo, Xiaochun Yang

**Affiliations:** 1 Institute of Pharmacology & Toxicology, College of Pharmaceutical Sciences, Zhejiang University, Hangzhou 310058, China; 12219045@zju.edu.cn (Y.W.); yang924@zju.edu.cn (B.Y.); 2 Center for Drug Safety Evaluation and Research, College of Pharmaceutical Sciences, Zhejiang University, Hangzhou 310058, China; jbl0915@zju.edu.cn (J.L.); 12219075@zju.edu.cn (X.F.); qiaojunhe@zju.edu.cn (Q.H.); peihualuo@zju.edu.cn (P.L.); 3 Innovation Institute for Artificial Intelligence in Medicine of Zhejiang University, College of Pharmaceutical Sciences, Zhejiang University, Hangzhou 310058, China; 4 School of Medicine, Hangzhou City University, Hangzhou 310015, China; 5 Hangzhou Institute of Innovative Medicine, College of Pharmaceutical Sciences, Zhejiang University, Hangzhou 310058, China; 6 Nanhu Brain-Computer Interface Institute, Hangzhou 311100, China

**Keywords:** Bortezomib, cuproptosis, peripheral neurotoxicity, (−)-Epigallocatechin Gallate, neuroprotection

## Abstract

Bortezomib (BTZ), the first-generation proteasome inhibitor, has been approved for the treatment of relapsed, refractory, and newly diagnosed multiple myeloma. Despite its remarkable antitumor efficacy, BTZ treatment is severely limited by a high incidence of systemic adverse reactions, primarily due to its non-selective cytotoxicity toward rapidly dividing normal cells and its potent neurotoxic effects on peripheral neurons. Bortezomib-induced peripheral neurotoxicity (BIPN) manifests as neuropathic pain and sensory abnormalities, affecting up to 31% to 64% of patients and limiting BTZ’s clinical use. Currently, the underlying mechanisms of BIPN are poorly understood. To evaluate the effects of BTZ on the functions of peripheral nerves in mice, we administered an intraperitoneal injection treatment for four weeks. Results indicated that BIPN caused mechanical allodynia, gait abnormalities, and pathological changes in myelin and axons in mice. This study confirms that BTZ upregulates the expression of the activating transcription factor 3 (ATF3), which in turn mediates the increased expression of the copper transporter SLC31A1, causing dysregulation of intracellular copper ion homeostasis and subsequent copper accumulation, and ultimately inducing the development of peripheral neurotoxicity. Elevated intracellular copper concentration exerts a dual effect: it directly promotes the oligomerization of Dihydrolipoamide S-acetyltransferase (DLAT) and concurrently damages the iron–sulfur cluster protein ferredoxin 1 (FDX1), collectively triggering the onset of cuproptosis. Green tea has garnered attention for its rich content of catechins, with (−)-Epigallocatechin Gallate (EGCG) being the most abundant catechin present. This study uncovers the molecular mechanism by which EGCG inhibits BTZ-induced cuproptosis through targeted regulation of copper homeostasis. Analyses demonstrate that EGCG significantly downregulates the expression of the copper transporter SLC31A1, thereby effectively suppressing transmembrane influx of extracellular copper ions. This intervention markedly reduces intracellular copper overload, eliciting a dual regulatory effect: on one hand, the decreased copper concentration directly inhibits the oligomerization of DLAT; on the other hand, it effectively protects the iron–sulfur cluster protein FDX1 from damage. This study aims to systematically elucidate the molecular mechanisms underlying BIPN and to evaluate the therapeutic potential of EGCG in alleviating BIPN, offering a novel therapeutic strategy for the prevention and treatment of BIPN.

## 1. Introduction

Bortezomib, as a first-line therapeutic agent for multiple myeloma—one of the second most common blood cancers, accounting for approximately 1% of all cancer—significantly improves patient survival when combined with thalidomide [1,2]. However, BTZ treatment is often associated with peripheral neurotoxicity, with an incidence ranging from 31% to 64% [3]. This adverse effect has a severe impact on patients’ quality of life, reducing adherence and treatment efficacy [4]. Currently, the underlying mechanisms of BIPN have not been fully elucidated.

The peripheral nervous system is mainly composed of Schwann cells and neurons. Neurons serve as the basic functional units for signal transmission, whereas Schwann cells exert protective, supportive, and trophic functions by forming myelin sheaths and secreting neurotrophic factors, which are crucial for maintaining the integrity of peripheral nerves [5]. Schwann cells not only promote myelination but also facilitate nerve repair and regeneration, demonstrating efficacy in treating peripheral nerve injuries in animal models [6]. Meanwhile, an increasing number of studies have demonstrated that regulated cell death and cellular dysfunction play crucial roles in the pathogenesis of BIPN [7,8]. Multiple regulated cell death pathways exist in animal cells. Notably, cuproptosis constitutes a distinct and copper-dependent form of regulated cell death [9,10]. Copper (Cu) is an essential trace element for humans [11], playing a key catalytic role in various biological processes. Cellular copper levels are tightly controlled to maintain a relatively low concentration, as excessive accumulation can induce cytotoxicity and cell death; thus, copper uptake, distribution, and elimination are rigorously regulated [12]. Copper deficiency in mammals can cause neuronal degeneration, cognitive deficits, cardiac hypertrophy, and immune dysfunction [13,14]; conversely, copper overload exerts toxic effects on cellular metabolism, mediating a form of copper-dependent cell death known as cuproptosis [10]. This copper-dependent controlled cell death represents a newly discovered mechanism in human cells, distinct from other known cell death pathways [15].

EGCG, the principal polyphenolic component of green tea, exhibits notable neuroprotective effects [16]. According to research, single or multiple administrations of 1 μM EGCG significantly alleviate neuronal death [17]. Additionally, EGCG has been shown to mitigate neuropathic pain associated with diabetic peripheral neurotoxicity, indicating its potential therapeutic benefits [18]. Currently, the prevention and treatment of BIPN remain a significant clinical challenge due to incomplete mechanistic understanding and a lack of validated therapeutic strategies. The potential neuroprotective role of EGCG in alleviating BIPN remains unreported. This study aims to systematically elucidate the molecular mechanisms underlying BIPN and to evaluate the therapeutic potential of (−)-Epigallocatechin Gallate (EGCG) in alleviating BIPN.

## 2. Results

### 2.1. BTZ Induces Peripheral Neurotoxicity

**Figure 1:**
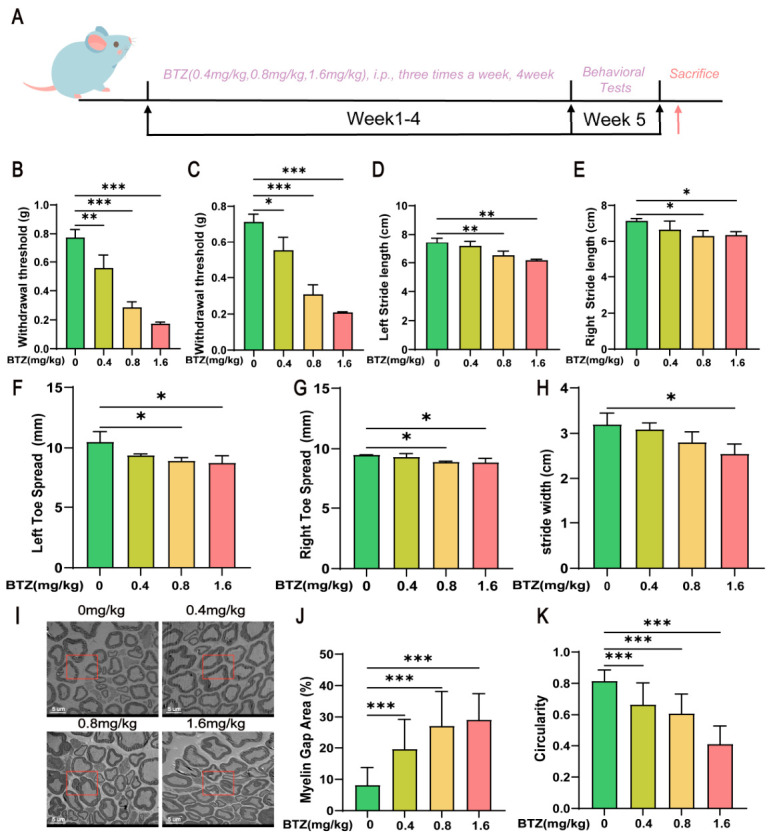
BTZ induces peripheral neurotoxicity. (**A**) The flowchart of the animal experiment. The changes in mechanical pain thresholds of the left (**B**) and right (**C**) hind paws of mice in each group following BTZ treatment. The effects of BTZ treatment on the stride length on the left (**D**) and right (**E**) sides. The impact of BTZ treatment on the toe spread distance of both hind paws on the left (**F**) and right (**G**) sides. Additionally, the influence of BTZ treatment on the overall stride width of mice in each group (**H**). (**I**) Representative transmission electron microscopy (TEM) images of sciatic nerves and semi-quantitative morphological analyses of (**J**) myelin gap area (%) and (**K**) myelin circularity. Scale bar, 5 μm. Data are expressed as mean ± standard deviation (SD), (*n* = 3 per group). *, *p* < 0.05; **, *p* < 0.01; ***, *p* < 0.001.

To investigate the effects of BTZ on peripheral nerves, we established an animal model to simulate the peripheral neurotoxicity induced by BTZ in clinical use. C57BL/6J mice were administered BTZ for four consecutive weeks, with three doses per week at concentrations of 0.4 mg/kg, 0.8 mg/kg, and 1.6 mg/kg for the model group. After four weeks of treatment, behavioral assessments were conducted to evaluate pain thresholds and gait coordination in the mice (Figure 1A). As shown in Figure 1B,C, BTZ significantly reduced the pain thresholds in both the left and right sides of the mice in a dose-dependent manner, indicating increased sensitivity to pain. Additionally, BTZ also dose-dependently decreased the stride length (Figure 1D,E), toe spread (Figure 1F,G), and stride width (Figure 1H) of the mice. Electron microscopy images of the sciatic nerve revealed that BTZ induced abnormalities in myelin (Figure 1I), characterized by abnormal folding of the myelin sheath, increased area of myelin gaps (Figure 1J), and reduced roundness (Figure 1K). In summary, those data demonstrate that BTZ significantly induces peripheral neurotoxicity.

### 2.2. Global Proteomic Analysis of BTZ-Induced Toxicity in RSC96: Mechanistic Investigation Based on Differential Protein Enrichment

**Figure 2:**
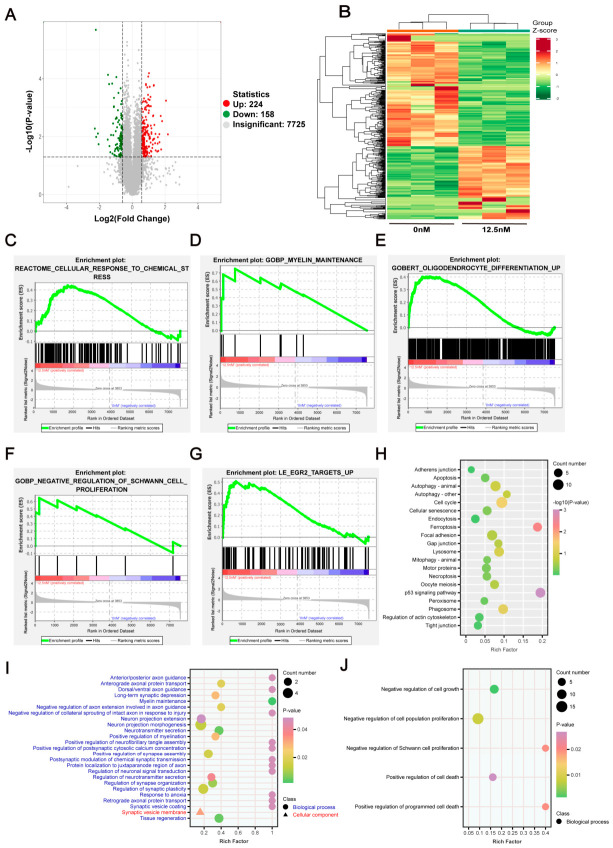
The negative impact of BTZ on the global protein pathway of RSC96. (**A**) A volcano plot depicts DEPs in RSC96 samples treated with BTZ. Red dots signify upregulated genes, green dots indicate downregulated genes, and gray dots represent non-significantly changed genes. (**B**) The heatmap of differential proteins from RSC96 treated with BTZ. After the RSC96 samples were treated with BTZ, pathway enrichment analysis via GSEA (**C**–**G**) was performed on all the identified proteins. KEGG (**H**) and GO (**I**,**J**) of the DEPs.

Schwann cells can effectively treat peripheral nerve injuries in animals by promoting myelin formation and nerve repair and regeneration [6]. To explore the potential mechanisms underlying BIPN, we treated RSC96 cells with BTZ and performed a 4D-DIA quantitative proteomic analysis. We identified a total of 8127 proteins in RSC96 following BTZ treatment, with 244 proteins upregulated and 158 proteins downregulated (Figure 2A). The expression of differentially expressed proteins (DEPs) is illustrated in the heatmap in Figure 2B. Subsequently, we conducted bioinformatics analyses to further investigate the pathways affected by BTZ. GSEA enrichment analysis of all identified proteins revealed that pathways related to chemical stress, myelin maintenance, and Schwann cell proliferation were enriched following BTZ treatment (Figure 2C–G). This indicates that BTZ exerts chemical toxicity on RSC96 and affects their proliferative capacity. Kyoto Encyclopedia of Genes and Genomes (KEGG) pathway analysis represents a comprehensive database that links genomic information to systematic biological functions and phenotypes [19]. Gene Ontology (GO) enrichment analysis is employed to annotate gene functions from three dimensions: biological process, cellular component, and molecular function [20]. Furthermore, KEGG pathway and GO enrichment analyses were performed. The KEGG results showed that pathways associated with cell death, including ferroptosis, autophagy, and apoptosis, were significantly enriched (Figure 2H), suggesting that BTZ may induce cell death. The GO enrichment results indicated that BTZ treatment promoted the enrichment of biological processes related to myelin maintenance, neuronal synapse extension, synaptic plasticity regulation, and tissue regeneration (Figure 2I), highlighting the toxic effects of BTZ on peripheral nerves and its impact on peripheral nerve function. Additionally, GO analysis revealed significant enrichment of pathways related to cell proliferation and death (Figure 2J). These analyses suggest that BIPN is closely associated with the proliferation and death of Schwann cells.

### 2.3. BTZ Induces Cuproptosis in Cells

**Figure 3:**
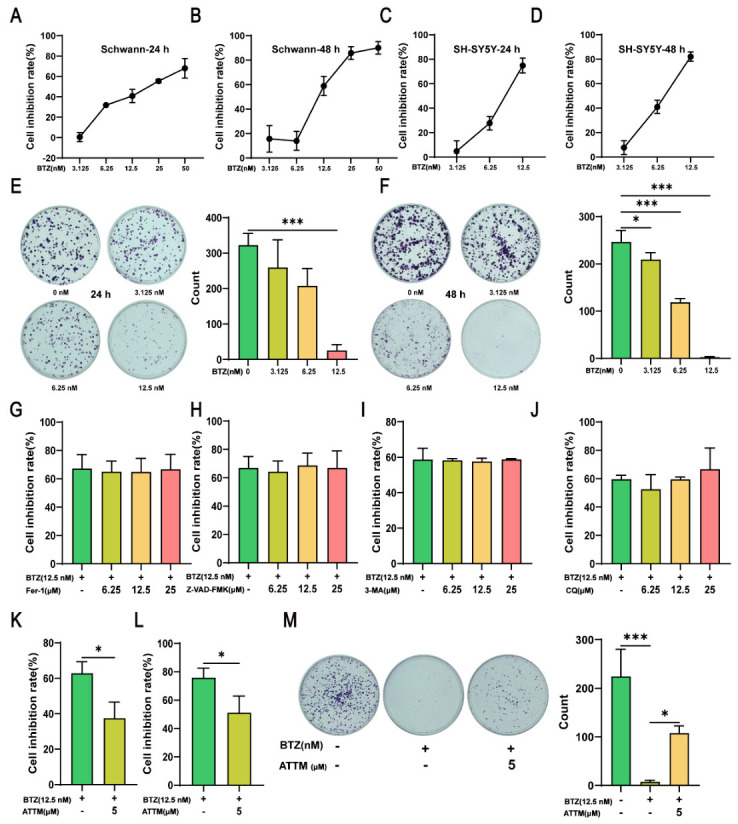
BTZ induces cuproptosis in cells. After treatment with BTZ: cell inhibition rate of RSC96 at 24 h (**A**) and 48 h (**B**) (*n* = 3); cell inhibition rate of SH-SY5Y at 24 h (**C**) and 48 h (**D**), detected via CCK-8 assay (*n* = 3). The colony formation of RSC96 at 24 h (**E**) and 48 h (**F**) after being treated with BTZ (*n* = 3). BTZ (12.5 nM) in combination with various concentrations of the ferroptosis inhibitor Fer-1 (**G**), the pan-caspase inhibitor Z-VAD-FMK (**H**) and two autophagy inhibitors, 3-MA (**I**) and CQ (**J**) after 48 h, with the cell inhibition rate of RSC96 detected via CCK-8 assay (*n* = 3). After treatment with BTZ (12.5 nM) in combination with the cuproptosis inhibitor ATTM for 48 h: cell inhibition rate of RSC96 (**K**) and SH-SY5Y (**L**) and the colony formation of RSC96 (**M**) (*n* = 3). Data are expressed as mean ± standard deviation (SD). *, *p* < 0.05; ***, *p* < 0.001.

After 24 and 48 h of BTZ treatment, we observed a concentration-dependent increase in cell death in RSC96 cells (Figure 3A,B) and SH-SY5Y cells (Figure 3C,D). Colony formation assays revealed that BTZ significantly reduced the proliferative capacity of RSC96 in a concentration-dependent manner at both 24 and 48 h (Figure 3E,F). These results indicate that BTZ increases cell death and affects cell proliferation in a concentration-dependent manner. Common forms of cell death include apoptosis, ferroptosis, and excessive autophagy [9]. The mechanism by which BTZ induces Schwann cell death remains unclear. To investigate the mode of cell death, we treated RSC96 cells with BTZ (12.5 nM) in combination with various concentrations of the pan-caspase inhibitor Z-VAD-FMK, the ferroptosis inhibitor Fer-1, and two autophagy inhibitors, CQ and 3-MA. Cell Counting Kit-8 (CCK-8) assays demonstrated that none of the four inhibitors effectively alleviated BTZ-induced cell death (Figure 3G–J), suggesting that BTZ may induce cell death through alternative mechanisms. Given the close relationship between cuproptosis and neuronal function [13,14], we treated the cells with the cuproptosis inhibitor ammonium tetrathiomolybdate (ATTM) alongside BTZ. CCK-8 results indicated that ATTM significantly inhibited cell death in both RSC96 (Figure 3K) and SH-SY5Y cells (Figure 3L), suggesting that BTZ may induce cell death via cuproptosis. The results of the RSC96 colony formation indicate that ATTM significantly inhibits the cell proliferation suppression induced by BTZ (Figure 3M).

### 2.4. BTZ Causes Cellular Dysfunction

**Figure 4:**
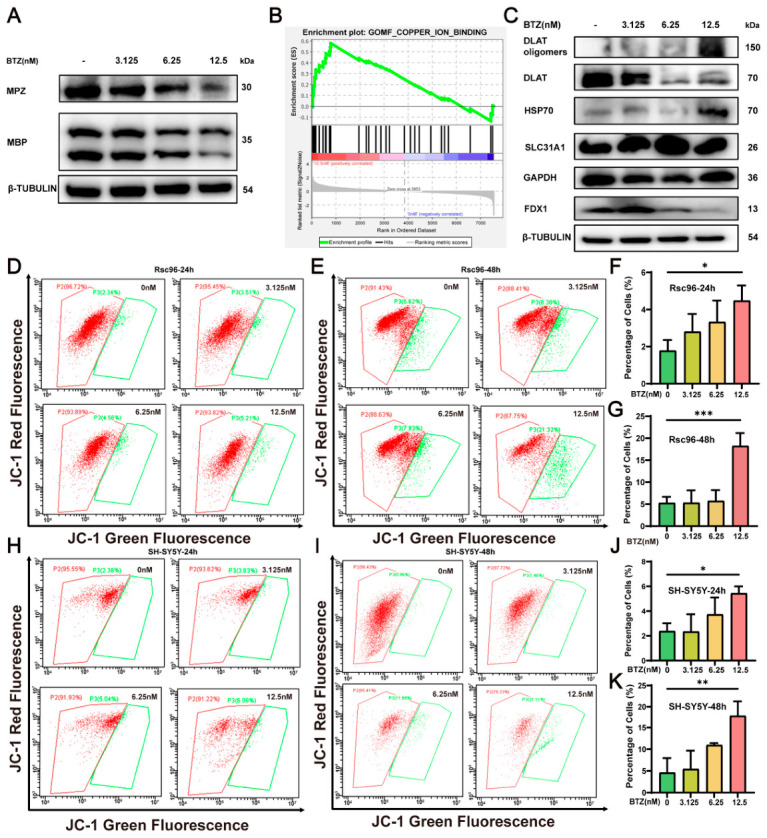
BTZ causes cellular dysfunction. (**A**) After BTZ 48 h treatment in RSC96, the protein expression levels of MPZ, β-TUBULIN and MBP in the RSC96 treated with BTZ were analyzed via Western blotting (*n* = 3). (**B**) After the RSC96 samples were treated with BTZ, pathway enrichment analysis via the GSEA was performed on cuproptosis-related proteins in all the identified proteins. (**C**) The protein expression levels of DLAT, DLAT oligomers, HSP70, SLC31A1, GAPDH, FDX1 and β-TUBULIN in the RSC96 treated with BTZ were analyzed via Western blotting (*n* = 3). The mitochondrial membrane potential in RSC96 treated with BTZ was analyzed by flow cytometry using the JC-1 probe for 24 h (**D**) and 48 h (**E**). Graph showing the changes in mitochondrial membrane potential at 24 h (**F**) and 48 h (**G**) (*n* = 3). The mitochondrial membrane potential in SH-SY5Y treated with BTZ was analyzed by flow cytometry using the JC-1 probe for 24 h (**H**) and 48 h (**I**). Graph showing the changes in mitochondrial membrane potential for 24 h (**J**) and 48 h (**K**) (*n* = 3). Data are expressed as mean ± standard deviation (SD). *, *p* < 0.05; **, *p* < 0.01; ***, *p* < 0.001.

We assessed the expression levels of myelin basic protein (MBP) and myelin protein zero (MPZ) in RSC96 (Figure 4A), finding that BTZ dose-dependently decreased the expression of MBP and MPZ, indicating that BTZ is toxic to the myelin of the sciatic nerve. Further GSEA enrichment analysis revealed significant enrichment of copper ion binding-related pathways following BTZ treatment (Figure 4B), along with notable enhancement of cell cycle-related pathways and mitochondrial function-related pathways (Figure S1A,B). To further elucidate the potential pathogenesis of BIPN, we hypothesized a connection between cuproptosis and BIPN, although the exact mechanism of this association remains unclear. Based on previous literature [21], we identified 347 differentially expressed genes related to copper (DE-CRGs) and found 11 common proteins in the intersection of DEGs and CRGs, including Alas1, etc. (Figure S1C). GO annotation of these 11 proteins revealed a close relationship with mitochondrial function (Figure S1D). The cell cycle is closely related to cell proliferation [22]. Flow cytometry results indicated that BTZ treatment induced cell cycle abnormalities in RSC96 (Figure S1E,F).

### 2.5. BTZ Induces Disruption of Intracellular Copper Homeostasis to Trigger Cuproptosis

**Figure 5:**
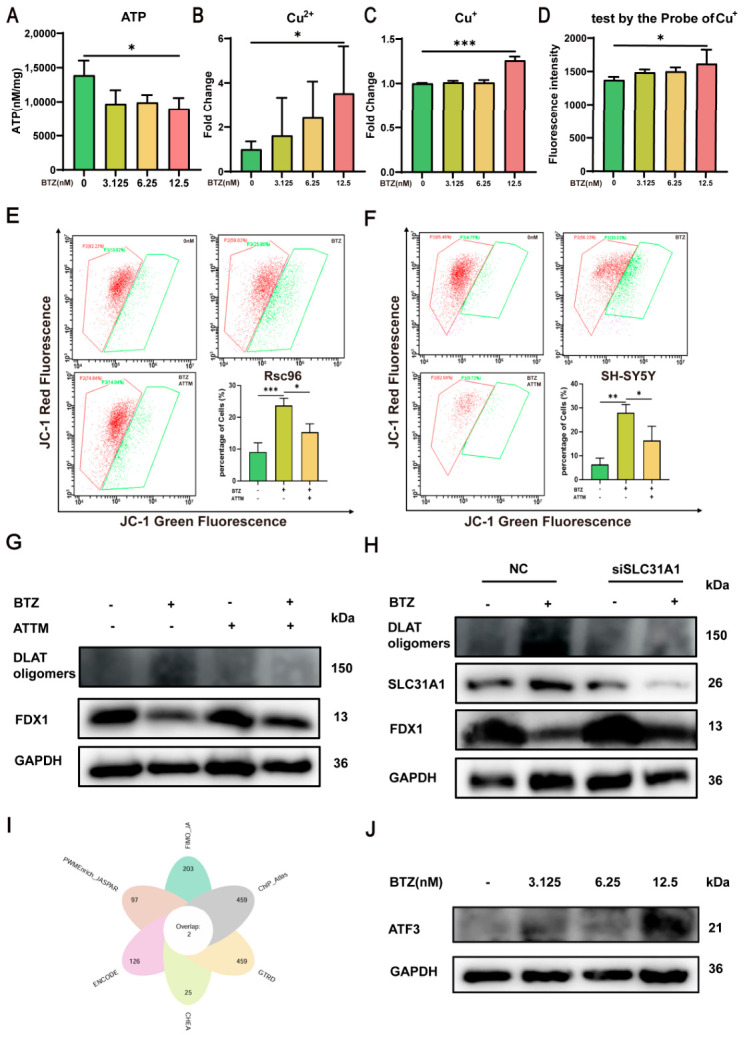
BTZ causes cuproptosis. (**A**) The measurement of ATP levels within RSC96 cells after BTZ treatment (*n* = 3). After BTZ treatment, the measurement of intracellular copper (**B**) and cuprous ion (**C**) concentrations and the measurement of the levels of cuprous ions in RSC96 cells using a copper ion probe (**D**) (*n* = 3). After treatment with BTZ (12.5 nM) in combination with ATTM, the mitochondrial membrane potential in RSC96 (**E**) and SH-SY5Y (**F**) was analyzed by flow cytometry using the JC-1 probe for 48 h (*n* = 3); (**G**) the protein expression levels of DLAT oligomers, FDX1 and GAPDH in RSC96. RSC96 cells were transfected with nontargeting siRNA (NC) or siRNA targeting SLC31A1 and then treated with or without BTZ for 48 h: (**H**) the protein expression levels of DLAT oligomers, SLC31A1, FDX1 and GAPDH in the RSC96 (*n* = 3). (**I**) The systematic prediction and analysis of potential transcription factor binding sites of the SLC31A1 gene using six transcription factor prediction databases: FIMO_JASPAR, PWMEnrich JASPAR, ENCODE, CHEA, GTRD, and ChIP-Atlas. (**J**) After a 48 h BTZ treatment in RSC96, the protein expression levels of ATF3 and GAPDH in the RSC96 treated with BTZ were analyzed via Western blotting (*n* = 3). Data are expressed as mean ± standard deviation (SD). *, *p* < 0.05; **, *p* < 0.01; ***, *p* < 0.001.

Subsequently, we assessed the protein expression levels of cuproptosis-related proteins DLAT, DLAT oligomers, HSP70, SLC31A1 and FDX1 (Figure 4C). After 48 h of treatment with varying concentrations of BTZ (0, 3.125, 6.25, and 12.5 nM), we observed a significant upregulation of DLAT oligomers, HSP70 and SLC31A1 expression levels, accompanied by a decrease in FDX1 and DLAT expression. DLAT, a key component of the pyruvate dehydrogenase complex (PDC), has been shown to bind intracellular copper ions, leading to its oligomerization and the formation of irreversible aggregates. These DLAT aggregates disrupt tricarboxylic acid (TCA) cycle function, thereby impairing ATP synthesis. Moreover, the formation of such aggregates triggers the loss of iron–sulfur cluster proteins, including FDX1. This cascade further exacerbates cellular energy deficits, thereby amplifying mitochondrial dysfunction [10]. The occurrence of cuproptosis is closely linked to mitochondrial function and copper ion levels [10]. During cuproptosis, cellular metabolic levels undergo significant changes, impacting mitochondrial function [13]. When the mitochondrial membrane potential is low, JC-1 exists in its monomeric form and cannot be retained within the mitochondrial matrix, producing green fluorescence. Changes in fluorescence color, combined with flow cytometry, enable effective detection of alterations in mitochondrial membrane potential [23]. Flow cytometry analysis demonstrated that BTZ dose-dependently reduced the mitochondrial membrane potential of RSC96 for 24 h and 48 h (Figure 4D–G) while also decreasing ATP levels (Figure 5A). At the same time, we found that BTZ could dose-dependently reduce the mitochondrial membrane potential of SH-SY5Y cells at 24 h and 48 h (Figure 4H–K). SLC31A1 can transport copper ions from the extracellular environment into the cell. The above results show that the expression of SLC31A1 has increased, and it may affect the copper ion content within the cell. Therefore, we measured the copper ion concentration within the cells. BTZ significantly increased intracellular copper and cuprous ion concentrations (Figure 5B,C). Using a cuprous ion probe, we further validated that BTZ significantly elevated the levels of cuprous ions in RSC96 cells (Figure 5D). ATTM is a copper ion chelator that can reduce the concentration of copper ions within cells [24]. We found that ATTM can elevate mitochondrial membrane potential in RSC96 and SH-SY5Y cells (Figure 5E,F). We observed that ATTM treatment markedly inhibited BTZ-induced DLAT oligomerization in RSC96 cells, while reducing damage to the iron–sulfur cluster protein FDX1 (Figure 5G). These findings further support the mechanism by which BTZ exerts cytotoxicity through the induction of copper-dependent cell death (cuproptosis). Collectively, our results suggest that upregulation of SLC31A1 promotes excessive copper influx into cells, leading to intracellular copper overload, which may serve as a key driver of BTZ-induced cuproptosis. Thus, we applied siRNA targeting SLC31A1 to silence the expression of the SLC31A1 gene. BTZ-induced upregulation of DLAT oligomers and SLC31A1, as well as downregulation of FDX1 (Figure 5H), were reversed by SLC31A1 silencing. This result indicates that suppression of SLC31A1 expression attenuates BTZ-induced cuproptosis, supporting that BTZ triggers cuproptosis via SLC31A1 upregulation. Collectively, these results demonstrate that 12.5 nM BTZ can induce cuproptosis. By integrating six transcription factor (TF) prediction databases (FIMO_JASPAR, PWMEnrich_JASPAR, ENCODE, CHEA, GTRD, and ChIP Atlas), we identified ATF3 and CREB1 as common upstream transcriptional regulators of SLC31A1 (Figure 5I). Notably, BTZ treatment only significantly upregulated ATF3 expression (Figure 5J). This suggests that BTZ may enhance SLC31A1 expression through ATF3-mediated transcriptional activation, subsequently triggering cuproptosis and contributing to the development of BIPN.

### 2.6. ATTM Mitigates BTZ-Induced Peripheral Neurotoxicity

**Figure 6:**
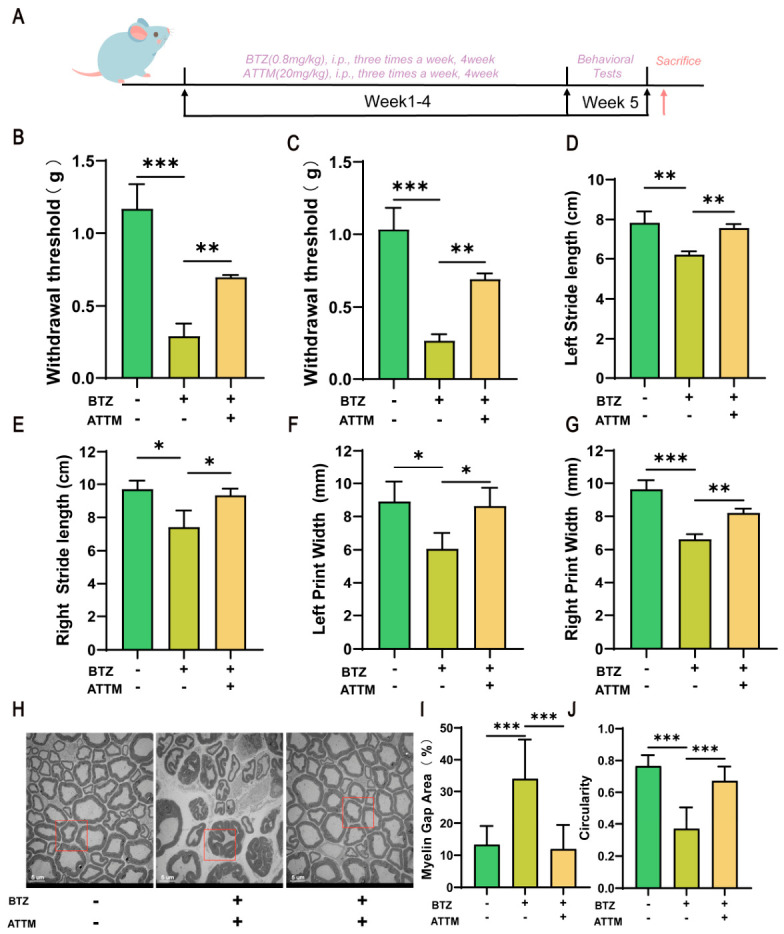
ATTM mitigates BTZ-induced peripheral neurotoxicity. (**A**) The flowchart of the animal experiment. After the combined treatment with BTZ and ATTM: changes in mechanical pain thresholds of the left (**B**) and right (**C**) hind paws of mice; the changes in stride length on the left (**D**) and right (**E**) sides; the changes in the toe spread distance of both hind paws on the left (**F**) and right (**G**) sides; After the combined treatment with BTZ and ATTM, (**H**) the representative TEM images of sciatic nerves and semi-quantitative morphological analyses of (**I**) myelin gap area (%) and (**J**) myelin circularity. Scale bar, 5 μm. Data are expressed as mean ± standard deviation (SD), (*n* = 3 per group). *, *p* < 0.05; **, *p* < 0.01; ***, *p* < 0.001.

To validate the effect of ATTM on BIPN in vivo, C57BL/6J mice were treated with 0.8 mg/kg of BTZ and 20 mg/kg of ATTM concurrently for four weeks, after which the pain threshold and gait behavior of the mice were assessed (Figure 6A). The results demonstrated that ATTM significantly increased the pain thresholds of both the left and right sides of the mice (Figure 6B,C), indicating its role in alleviating pain sensitivity. Additionally, ATTM also improved the stride length (Figure 6D,E) and toe spread (Figure 6F,G) of the mice. Electron microscopy revealed that ATTM improved the BTZ-induced abnormalities in the sciatic nerve myelin sheath (Figure 6H), significantly reducing myelin folding deformities, decreasing the area of myelin gaps (Figure 6I) and increasing myelin circularity (Figure 6J). In summary, these data demonstrate that ATTM effectively ameliorates BIPN, further supporting that BTZ induces peripheral neurotoxicity in mice through the mechanism of cuproptosis.

### 2.7. EGCG Reverses BTZ-Induced Cuproptosis

**Figure 7:**
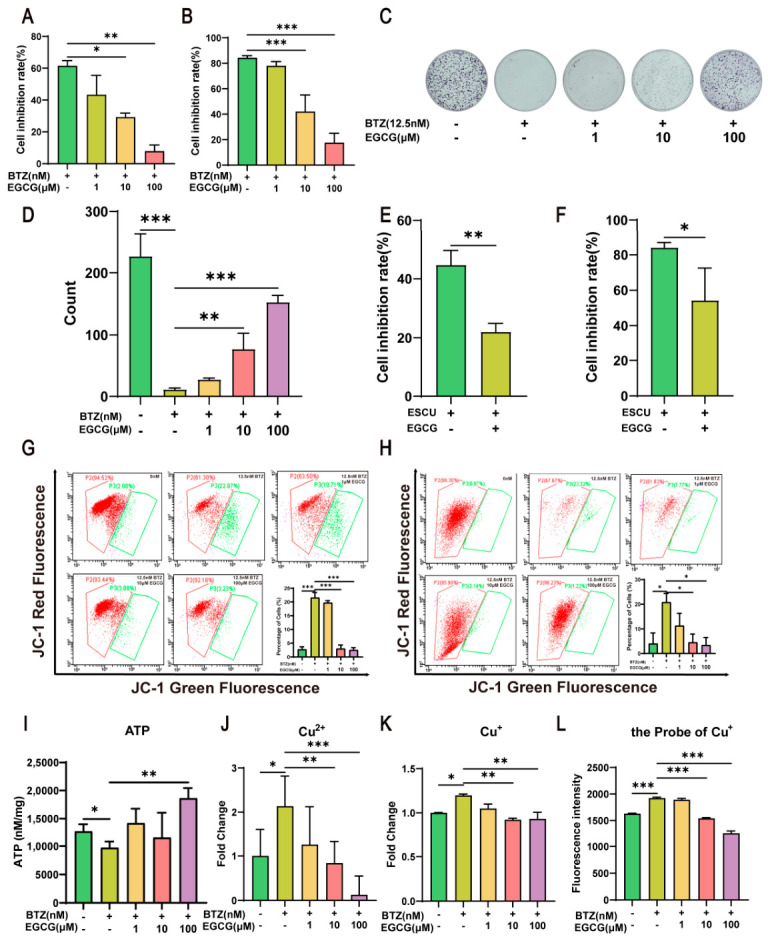
EGCG reverses BTZ-induced cuproptosis and cellular dysfunction. After combined treatment with BTZ and EGCG for 48 h, the cell inhibition rate of RSC96 (**A**) and SH-SY5Y (**B**) was detected via CCK-8 assay (*n* = 3); (**C**) the colony formation of RSC96; and (**D**) graph showing the changes in colony formation (*n* = 3). After the combined treatment with BTZ and cuproptosis-inducer elesclomol + CuCl_2_ (ESCU) for 48 h, the cell inhibition rate of RSC96 (**E**) and SH-SY5Y (**F**) was detected via CCK-8 assay (*n* = 3). After the combined treatment with BTZ and EGCG for 48 h, the mitochondrial membrane potential in RSC96 (**G**) and SH-SY5Y (**H**) was analyzed by flow cytometry using the JC-1 probe and graph showing the changes in mitochondrial membrane potential (*n* = 3); (**I**) the measurement of ATP levels within RSC96 cells; the measurement of intracellular copper (**J**) and cuprous ion (**K**) concentrations and the measurement of the levels of cuprous ions in RSC96 cells using a copper ion probe (**L**) (*n* = 3). Data are expressed as mean ± standard deviation (SD). *, *p* < 0.05; **, *p* < 0.01; ***, *p* < 0.001.

The survival of Schwann cells plays a crucial role in the functionality of peripheral nerves [6]. Therefore, investigating strategies to mitigate BTZ-induced cuproptosis is of significant importance. Previous studies have demonstrated that various herbal monomers, including Glycyrrhizic acid [25] and EGCG [26], exhibit neuroprotective effects; this indicates the potential of single compounds from traditional Chinese medicine in treating peripheral neurotoxicity. We found that numerous natural monomers derived from traditional Chinese medicine exert potential neuroprotective effects. Accordingly, we established a dedicated compound library containing 89 monomers from traditional Chinese medicine. By screening the traditional Chinese medicine monomer compound library (Figure S2A–C), it was found that EGCG could significantly reduce cell death caused by BTZ, and this effect was dose-dependent (Figure 7A). Moreover, EGCG could also effectively alleviate the death of SH-SY5Y cells in a dose-dependent manner (Figure 7B). To assess the impact of EGCG on the proliferation capacity of RSC96 cells, a colony formation assay was performed (Figure 7C,D), revealing that EGCG significantly promoted RSC96 proliferation in a dose-dependent manner. Elesclomol, a copper ion carrier, can enhance the uptake of copper ions in cells by binding with CuCl_2_, thereby increasing intracellular copper concentrations and inducing cuproptosis [27]. Treatment of RSC96 cells with 12.5 μM Elesclomol + CuCl_2_ (ESCU) for 48 h induced cuproptosis, while co-treatment with EGCG (Figure 7E) significantly reduced the incidence of cuproptosis. A similar protective effect was observed in SH-SY5Y cells (Figure 7F), indicating that EGCG can inhibit cell death induced by copper carriers.

### 2.8. EGCG Inhibits Cuproptosis by Restoring the Copper Balance Within Cells

**Figure 8:**
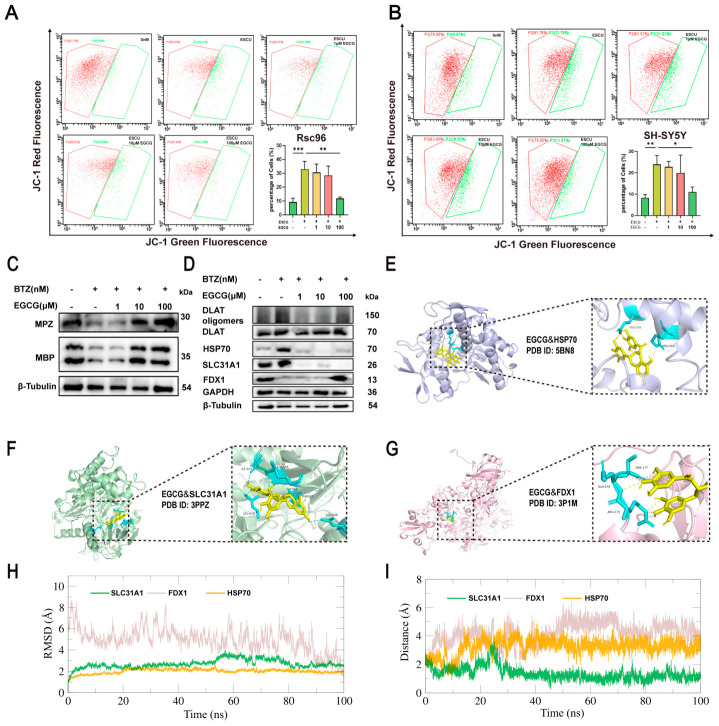
EGCG targets SLC31A1 to reverse copper-induced death and mitochondrial dysfunction. After treatment with ESCU (12.5 μM) in combination with EGCG, the mitochondrial membrane potential in RSC96 (**A**) and SH-SY5Y (**B**) was analyzed by flow cytometry using the JC-1 probe for 48 h (*n* = 3). After combined treatment with BTZ and EGCG for 48 h, (**C**) the protein expression levels of MPZ, MBP and β-TUBULIN in the RSC96 treated with BTZ were analyzed via Western blotting (*n* = 3); (**D**) the protein expression levels of DLAT, DLAT oligomers, HSP70, SLC31A1, FDX1, GAPDH and β-TUBULIN in the RSC96 treated with BTZ were analyzed via Western blotting (*n* = 3). (**E**–**G**) Molecular interactions between (**E**) EGCG and HSP70, (**F**) EGCG and SLC31A1, (**G**) EGCG and FDX1. (**H**) RMSD of the protein of 3 proteins in a 100 ns MD simulation. (**I**) The center-of-geometry distance between the 3 proteins and the small molecule. Data are expressed as mean ± standard deviation (SD). *, *p* < 0.05; **, *p* < 0.01; ***, *p* < 0.001.

Flow cytometry results indicated that EGCG dose-dependently elevated the mitochondrial membrane potential of RSC96 cells (Figure 7G) and SH-SY5Y cells (Figure 7H), restoring them to control levels, while also increasing ATP levels (Figure 7I). Meanwhile, EGCG can elevate the mitochondrial membrane potential that was reduced by ESCU in RSC96 and SH-SY5Y cells (Figure 8A,B). Copper ions can trigger proteotoxic stress, leading to cell death [10], making it crucial to assess whether EGCG reduces intracellular copper ion levels. We measured the copper ion content in RSC96 cells, and as illustrated in Figure 7J,K, EGCG was found to decrease the levels of intracellular copper and cuprous ions. Additionally, we employed a cuprous ion probe to detect cuprous ion levels in RSC96 cells (Figure 7L), revealing that EGCG significantly decreased the levels of cuprous ions within these cells. Collectively, these data suggest that EGCG may inhibit cuproptosis by reducing the concentration of copper ions within the cells. Immunoblotting analysis demonstrated that after the combined treatment with BTZ and EGCG for 48 h, EGCG dose-dependently reversed the reduction in MBP and MPZ protein expression following BTZ treatment in RSC96 (Figure 8C), indicating its protective effect on myelin integrity. Furthermore, EGCG significantly downregulated the expression of cuproptosis-related proteins DLAT, DLAT oligomers, HSP70, and SLC31A1 while upregulating FDX1 expression (Figure 8D). Molecular docking studies using AutoDock Vina revealed strong binding affinities of EGCG with HSP70, SLC31A1, and FDX1 proteins (docking scores of −8.1, −10.8, and −7.0 kcal/mol, respectively). Structural visualization indicated stable binding at the protein interface (Figure 8E–G). In summary, EGCG inhibits cuproptosis by regulating copper ion homeostasis and directly interacting with cuproptosis-related proteins. The results of the molecular dynamics (MD) simulation are shown in Figure 8H,I. As shown in Figure 8H, the root mean square deviation (RMSD) of the protein remains stable within 100 nanoseconds. The root mean square deviation value indicates that the protein maintains a stable conformation during the binding process. Similarly, in Figure 8I, the geometric center distance between the protein and the small molecule is given. This distance remains stable throughout the simulation and fluctuates very little, indicating the presence of a strong and persistent binding interaction throughout the simulation. These results collectively suggest that under the simulation conditions, the protein structure and its interaction with the small molecule are highly stable, providing a reliable basis for further functional and mechanism studies. Furthermore, as shown in Figure S2D, EGCG was able to reverse the cell cycle abnormalities induced by BTZ. This study uncovers the molecular mechanism by which EGCG inhibits BTZ-induced cuproptosis through targeted regulation of copper homeostasis. Mechanistic analyses demonstrate that EGCG significantly downregulates the expression of the copper transporter SLC31A1, thereby effectively suppressing the transmembrane influx of extracellular copper ions. This intervention markedly reduces intracellular copper overload, eliciting a dual regulatory effect: on one hand, the decreased copper concentration directly inhibits the oligomerization of DLAT; on the other hand, it effectively protects the iron–sulfur cluster protein FDX1 from damage. Based on the CCK-8 assay results, the presence of EGCG did not significantly alter the inhibitory effect of BTZ on Mino cells, suggesting that EGCG does not affect BTZ-mediated cytotoxicity (Figure S2E).

### 2.9. EGCG Mitigates BTZ-Induced Peripheral Neurotoxicity

**Figure 9:**
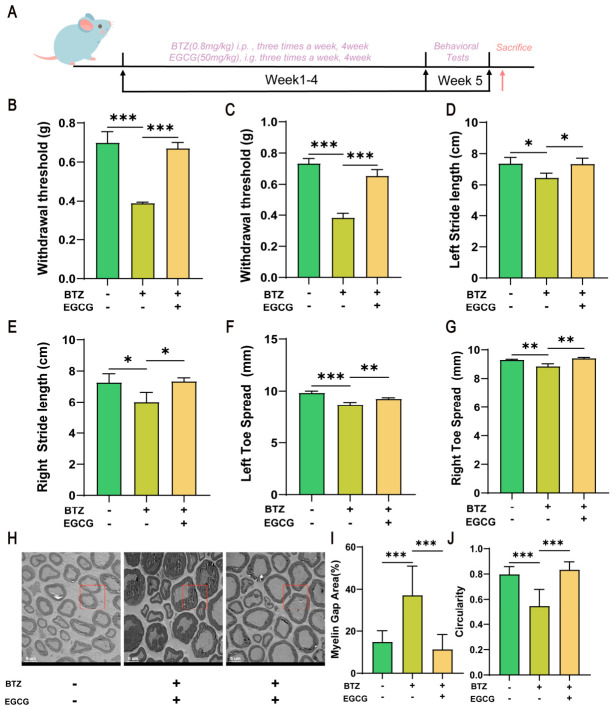
EGCG mitigates BTZ-induced peripheral neurotoxicity. (**A**) The flowchart of the animal experiment. After the combined treatment with BTZ and EGCG: changes in mechanical pain thresholds of the left (**B**) and right (**C**) hind paws of mice; changes in stride length on the left (**D**) and right (**E**) sides; changes in the toe spread distance of both hind paws on the left (**F**) and right (**G**) sides; After the combined treatment with BTZ and EGCG: (**H**) representative TEM images of sciatic nerves and semi-quantitative morphological analyses of (**I**) myelin gap area (%) and (**J**) myelin circularity. Scale bar, 5 μm. Data are expressed as mean ± standard deviation (SD) (*n* = 3 per group). *, *p* < 0.05; **, *p* < 0.01; ***, *p* < 0.001.

To validate the effect of EGCG on BIPN in vivo, C57BL/6J mice were treated with 0.8 mg/kg of BTZ and 50 mg/kg of EGCG concurrently for four weeks, after which the pain threshold and gait behavior of the mice were assessed (Figure 9A). The results demonstrated that EGCG significantly increased the pain thresholds of both the left and right sides of the mice in a dose-dependent manner (Figure 9B,C), indicating its role in alleviating pain sensitivity. Additionally, EGCG also dose-dependently improved the stride length (Figure 9D,E) and toe spread (Figure 9F,G) of the mice. Electron microscopy revealed that EGCG improved the BTZ-induced abnormalities in the sciatic nerve myelin sheath (Figure 9H), significantly reducing myelin folding deformities, decreasing the area of myelin gaps (Figure 9I) and increasing myelin circularity (Figure 9J). Collectively, those data support the conclusion that EGCG exerts a significant protective effect in ameliorating BIPN.

## 3. Discussion

The incidence and mortality rates of multiple myeloma remain high, and BTZ, as a first-line treatment, has been shown to significantly improve patient survival [1,2]. However, the clinical application of BTZ is inevitably associated with various side effects, among which peripheral neurotoxicity is particularly prominent, with an incidence rate ranging from 31% to 64% [3]. BIPN primarily manifests as moderate to severe neuropathic pain in the distal extremities [28], with some patients experiencing motor impairments such as muscle weakness and atrophy [29]. In this study, we established a BIPN animal model and found that BTZ significantly reduced the pain thresholds of mice, thereby enhancing their pain sensitivity. Additionally, BTZ led to a decrease in stride length and toe spread, reflecting impaired motor function. Furthermore, BTZ caused a reduction in myelin circularity, an expansion of myelin gaps, and abnormal myelin folding, indicating structural damage to the myelin sheath.

The mechanisms underlying BIPN are not yet fully understood. GSEA and GO enrichment analyses revealed that BTZ interferes with pathways related to myelin maintenance, Schwann cell proliferation, and synaptic plasticity, while also affecting tissue regeneration processes. KEGG enrichment analysis showed significant activation of cell death-related signaling pathways, including ferroptosis, autophagy, and apoptosis. Our study provides the first evidence that BTZ not only induces cuproptosis in RSC96 but also leads to cuproptosis in the SH-SY5Y cells. Further observations indicated that BTZ treatment resulted in abnormal changes in the cell cycle of RSC96. Schwann cells possess regenerative capabilities and play a crucial role in myelination, as well as promoting nerve repair and regeneration, highlighting their potential application in the repair of peripheral nerve injuries [6].

Copper deficiency can lead to neurodegeneration in mammals [13,14], while copper ion overload can also be toxic to cells, resulting in cuproptosis [10]. This study uncovers a novel molecular mechanism underlying BTZ-induced peripheral neurotoxicity, centered on cuproptosis mediated by disrupted copper homeostasis. Our data reveal that BTZ significantly upregulates ATF3 expression, which transcriptionally activates SLC31A1, thereby enhancing intracellular copper influx. This dysregulation leads to pathological copper accumulation and ultimately triggers cuproptosis. Gene set enrichment analysis revealed significant enrichment of copper-binding pathways, implicating copper homeostasis disruption as a pivotal factor in BIPN. Excess intracellular copper mediates cellular injury via dual mechanisms: directly promoting the aggregation of the DLAT protein and impairing the iron–sulfur cluster protein FDX1, which synergistically trigger cuproptosis. The abnormal accumulation of copper ions can disrupt various cellular processes, leading to mitochondrial dysfunction [10]. Additionally, previous studies have shown that BTZ damages mitochondrial function in the peripheral nervous system [30]. Moreover, BTZ substantially decreases mitochondrial membrane potential and ATP levels in RSC96 cells, confirming mitochondrial dysfunction.

We demonstrated that ATTM restores mitochondrial membrane potential and ATP content in RSC96 and SH-SY5Y cells, indicating that chelation of intracellular copper mitigates copper overload and significantly rescues cellular function. ATTM also reverses BTZ-induced DLAT oligomerization and alleviates FDX1 damage in RSC96 cells. In addition, ATTM was able to increase the pain threshold, rescue gait coordination, and markedly improve axonal pathological alterations in mice. Collectively, these findings corroborate the notion that BTZ triggers peripheral neurotoxicity through a cuproptosis-dependent mechanism. Collectively, our results indicate that upregulation of SLC31A1 leads to excessive copper uptake, causing intracellular copper overload that likely drives BTZ-induced cuproptosis. Consistently, silencing SLC31A1 via siRNA abrogated BTZ-induced DLAT oligomerization and iron–sulfur cluster protein FDX1 disruption, suggesting that suppression of SLC31A1 expression rescues cells from cuproptosis and corroborates the hypothesis that BTZ induces cuproptosis through SLC31A1 upregulation. In summary, these data reveal that BTZ triggers peripheral neurotoxicity by inducing intracellular copper imbalance and subsequent cuproptosis. This mechanism not only expands the molecular understanding of BTZ neurotoxicity but also identifies potential therapeutic targets for BTZ-induced peripheral neurotoxicity.

BIPN severely impairs the quality of life of patients, yet effective treatment and prevention strategies remain lacking [31]. Current therapies primarily focus on symptomatic interventions for peripheral neuropathic pain, including opioids and non-steroidal anti-inflammatory drugs, which do not provide a true cure [32]. EGCG, a rich active component in green tea, has neuroprotective effects [16]. Previous studies have shown that EGCG can have beneficial effects on diabetic peripheral neurotoxicity in rats [18], but whether EGCG can alleviate BIPN has not been reported. When EGCG was administered concurrently with BTZ in mice, we found that EGCG could reverse the decrease in pain thresholds and motor function impairments caused by BTZ. Moreover, EGCG reversed the abnormal myelin folding and reduced axonal roundness induced by BTZ, suggesting that EGCG has the potential to protect their myelin structure, thereby exerting neuroprotective effects. In terms of functional improvement, EGCG demonstrated a mitigating effect on BTZ-related peripheral nerve damage, supporting its potential as an intervention strategy.

Previous studies have indicated that EGCG significantly reduces the death of PC12 cells induced by neurotoxins [17]. Our study found that EGCG reduced the mortality of BTZ-induced RSC96 and SH-SY5Y cells, improving the abnormal cell cycle. Our findings demonstrate that EGCG significantly alleviates BTZ-induced mitochondrial dysfunction and peripheral neurotoxicity by inhibiting the occurrence of cuproptosis. Specifically, EGCG markedly increased intracellular ATP levels and enhanced mitochondrial membrane potential, indicating its critical role in restoring mitochondrial function. Moreover, EGCG significantly downregulated the expression of the copper transporter SLC31A1 and reduced aberrant intracellular copper accumulation, supporting its potential role in regulating copper homeostasis. Functionally, EGCG effectively reversed BTZ-induced oligomerization of DLAT in RSC96 cells and mitigated damage to the iron–sulfur cluster protein FDX1, further elucidating its ability to inhibit cuproptosis. Concurrently, EGCG promoted the recovery of MBP and MPZ expression in Schwann cells, suggesting a potential effect on nerve myelin repair. Elesclomol + CuCl_2_ can induce cuproptosis in RSC96 cells [27]. Utilizing an established model of cuproptosis induced by combined Elesclomol and CuCl_2_ treatment in RSC96 cells, we found that EGCG significantly suppressed cuproptosis and attenuated the loss of mitochondrial membrane potential; consistent results were observed in SH-SY5Y cells. Collectively, these data indicate that EGCG may mitigate BTZ-induced peripheral neurotoxicity through inhibition of cuproptosis. These findings not only deepen our understanding of the mechanisms underlying BTZ-associated neurotoxicity but also highlight EGCG as a promising therapeutic strategy targeting cuproptosis for the treatment of BIPN.

## 4. Materials and Methods

### 4.1. Animal Study and Treatment

Adult male C57BL/6J mice weighing approximately 25 g were obtained from Beijing Vital River Laboratory Animal Technology Co., Ltd. (Beijing, China). This experiment was approved by the ethics committee of experimental animals of Innovation Institute for Artificial Intelligence in Medicine of Zhejiang University (Animal Experiment Ethics Review No. DW202404161604). The ethical approval was obtained on 16 April 2024. Prior to experimentation, animals were housed in a controlled SPF environment with ambient temperature maintained between 22 °C and 25 °C, relative humidity of 40–70%, a 12 h light/dark cycle, adequate ventilation, and ad libitum access to food and water. Mice were randomly assigned to experimental groups and individually housed in metabolic cages. A one-week acclimation period was observed before behavioral testing. For sample collection, mice were fasted for 12 h, anesthetized with sodium pentobarbital (45 mg/kg), and euthanized in a fully sedated state. Firstly, we established a BIPN model in vivo using C57BL/6J mice as the host. We then evaluated the peripheral neurotoxicity of BTZ in mice at the animal level, providing a model basis for subsequent studies on toxic mechanisms and intervention strategies. According to previous studies [33], BTZ was administered via intraperitoneal injection at doses of 0.4 mg/kg, 0.8 mg/kg, and 1.6 mg/kg, three times a week for 4 consecutive weeks. To evaluate the therapeutic potential of ATTM against BIPN, mice received co-treatment with 0.8 mg/kg BTZ (administered intraperitoneally) and 20 mg/kg ATTM (administered intraperitoneally) for four weeks, with administration three times weekly (each group *n* = 5–8). To evaluate the therapeutic potential of EGCG against BIPN, mice received co-treatment with 0.8 mg/kg. According to previous studies [26], mice were treated with BTZ (administered intraperitoneally) and 50 mg/kg EGCG (administered orally), dissolved in physiological saline, for four weeks, with administration three times weekly (each group *n* = 5–8). Efforts were made to alleviate the suffering of the animals as much as possible and to minimize the number of animals needed.

### 4.2. Cell Culture

The RSC96 (ATCC: CRL-2765) represents a commonly utilized rat Schwann cell line, and the SH-SY5Y (ATCC: CRL-2266) is a type of human neuroblastoma cell, which serves as a widely adopted cell model in neural research [34,35,36,37,38,39]. The Mino (ATCC: CRL-3000) is a human peripheral blood-derived mantle cell lymphoma cell line. RSC96 and SH-SY5Y cell lines were bought from the Procell Life Science & Technology Co., Ltd. (Wuhan, China) with an STR document, and cultured in DMEM media (10569010, Gibco, New York, NY, USA) supplemented with 10% fetal bovine serum (16140071, Gibco, New York, NY, USA) and 1% penicillin/streptomycin. The Mino cell lines were bought from the Procell Life Science & Technology Co., Ltd. with an STR document and cultured in RPMI-1640 (11875093, Gibco, New York, NY, USA) + 20% fetal bovine serum (16140071, Gibco, New York, NY, USA) + 1% penicillin/streptomycin, which were all in a 37 °C and 5% CO_2_ incubator.

### 4.3. Drug

Bortezomib, the ferroptosis inhibitor (Ferrostatin1, Fer-1), EGCG, the cuproptosis inducer Elesclomol, copper chloride were purchased from Yuanye Biotechnology Co., Ltd. (Shanghai, China). The pan-caspase inhibitor (Z-VAD-FMK) was purchased from Selleck Chemicals (Shanghai, China). The cuproptosis inhibitor ATTM was purchased from MedChemExpress (MCE) (Shanghai, China). Autophagy inhibitors (Chloroquine, CQ; 3-Methyladenine, 3-MA) were purchased from Shanghai TargetMol Biotechnology Co., Ltd. (Shanghai, China).

In some experiments, the cells were co-treated with BTZ and several inhibitors including Fer-1, ATTM, Z-VAD-FMK, CQ, and 3-MA at concentrations of 3.125, 6.25, and 12.5 μM, respectively, or EGCG at concentrations of 1, 10, and 100 μM for 48 h. The copper ionophore Elesclomol was mixed with copper chloride at a 1:1 molar ratio, and the mixture (with each component at a final concentration of 12.5 μM) was then co-incubated with target cells for 48 h to induce cuproptosis in the cells.

The traditional Chinese medicine monomer compound library established by the research group (a total of 89 compounds) was purchased from Shanghai TargetMol Biotechnology Co., Ltd.

The traditional Chinese medicine monomeric compounds used in the present study are listed below, with a total of 89 compounds: 1. (−)-Epigallocatechin Gallate, 2. Isoliquiritigenin, 3. Glycyrrhizic acid, 4. Licoisoflavone A, 5. Lupiwighteone, 6. Astragalus polysaccharide, 7. Berberine, 8. Obacunone, 9. 8-Oxoepiberberine, 10. *N*-trans-Feruloyltyramine, 11. Wogonoside, 12. Skullcapflavone II, 13. Salvigenin, 14. Moslosooflavone, 15. Norwogonin, 16. Rhodiosin, 17. Tyrosol, 18. Skimmin, 19. Ellagic acid, 20. Chrysoeriol, 21. Ethyl linoleate, 22. Cyclopamine, 23. Isoverticine, 24. Arctiin, 25. Squalene, 26. Linarin, 27. Aloe emodin, 28. Genkwanin, 29. Diosmetin, 30. Isoferulic acid, 31. Tangeretin, 32. Hesperetin, 33. 7-Hydroxycoumarin, 34. Myrcene, 35. Caryophyllene, 36. Methylophiopogonanone A, 37. Daucosterol, 38. Vanillic Acid, 39. Kaempferol, 40. Kumatakenin, 41. Formononetin, 42. Beta-Sitosterol, 43. Coptisine, 44. Epiberberine, 45. Acacetin, 46. Atractylenolide I, 47. Atractyloside A, 48. Saikosaponin A, 49. Nootkatone, 50. Saikosaponin b3, 51. Senkyunolide I, 52. Torachrysone-8-O-b-D-glucoside, 53. Desoxyrhaponticin, 54. Rhein 8-Glucoside, 55. Glycitin, 56. Genistin, 57. Angeloylgomisin H, 58. Nudifloric Acid, 59. Glabridin, 60. Glycyrol, 61. Liquiritin, 62. 18β-Glycyrrhetinic acid, 63. Kakkalide, 64. Puerarin, 65. Fucoxanthin, 66. Crocin, 67. Safranal, 68. Picroside I, 69. Astragaloside, 70. Cyclogalegenin, 71. Pterostilbene, 72. Baicalein, 73. Wogonin, 74. Viscidulin III, 75. Gypenoside XLIX, 76. gypenoside A, 77. macranthoidin A, 78. Platicodigenin, 79. Matrine, 80. Amygdalin, 81. D-(−)-Mandelic acid, 82. Ganoderic acid H, 83. Isorhapontigenin, 84. Crustecdysone, 85. Panaxadiol, 86. Ginsenoside Rk3, 87. Ginsenoside Re, 88. Ginsenoside Rb2, 89. Cinnamyl alcohol.

### 4.4. Proteomics

RSC96 cells (*n* = 3) were collected for proteomic analysis. The samples were processed by Wuhan Maiwei Metabolic Biotechnology Co., Ltd. (Wuhan, China) using 4D-DIA quantitative proteomics. Following protein extraction, total protein concentrations were determined using the BCA assay, and samples were desalted via C18 spin columns prior to liquid chromatography-tandem mass spectrometry (LC-MS/MS) analysis. Raw data were analyzed using DIA-NN (version 1.8.1), with spectral libraries generated from the UniProt proteome database. False discovery rate (FDR) was controlled at less than 1% at both the protein and peptide ion levels, and remaining identifications were used for subsequent quantitative analysis. Differentially expressed proteins were identified with the criteria of fold change (FC) ≥ 1.5 or ≤0.6667, and *p* < 0.05.

The mass spectrometry proteomics data have been deposited to the ProteomeXchange Consortium (https://proteomecentral.proteomexchange.org, accessed on 21 January 2026) via the iProX partner repository [40,41] with the dataset identifier PXD072911.

### 4.5. CCK-8

The cell inhibition rate was assessed using the CCK-8 (Catalog No. C0005, TargetMol). Following the removal of the existing culture medium, fresh medium containing 10% CCK-8 reagent was added. Cells were incubated at 37 °C in the dark for 60 min, and absorbance was measured at 450 nm using a TECAN multimode microplate reader (Tecan, Männedorf, Switzerland). The cell inhibition rate was calculated as: [(control group − experimental group)/(control group − blank control)] × 100%.

### 4.6. Colony Formation

Cells were plated in cell culture dishes at a density of approximately 1000 cells per well. Following drug treatment and removal of the culture medium, fresh medium was added, and the cells were cultured until colonies appeared, which took about one week. After fixing with cold methanol for 15 min, the cells were stained with crystal violet and photographed.

### 4.7. JC-1 Assays

The changes in mitochondrial membrane potential in RSC96 cells were assessed using a mitochondrial membrane potential detection kit (Beyotime Biotechnology, Shanghai, China, C2005). JC-1 is an excellent fluorescent probe for detecting mitochondrial membrane potential. When the mitochondrial membrane potential is high, JC-1 forms aggregates that accumulate in the mitochondrial matrix, emitting red fluorescence. Conversely, when the mitochondrial membrane potential is low, JC-1 exists in a monomeric form and fails to accumulate in the mitochondrial matrix, producing green fluorescence. By monitoring these fluorescence shifts and utilizing flow cytometry, changes in mitochondrial membrane potential can be effectively assessed. Briefly, drug-treated RSC96 cells were digested with trypsin, washed with 1× PBS, and then incubated with 1× JC-1 staining working solution. After a 20 min incubation at 37 °C in the dark, the cells were washed with pre-cooled 1× staining buffer. Data acquisition and analysis were performed within 5 min using a BECKMAN COULTER flow cytometer (Beckman Coulter, Inc., Brea, CA, USA).

### 4.8. Cell Cycle Analysis

The changes in the cell cycle of RSC96 cells were evaluated using a cell cycle kit (Beyotime Biotechnology, C1052). In brief, cells were washed with 1× PBS and fixed overnight at 4 °C with pre-cooled 70% ethanol. A 1× propidium iodide staining solution was prepared and supplemented with RNase A, followed by a 30 min incubation at 37 °C in the dark. Data acquisition and analysis were conducted within 5 min using a BECKMAN COULTER flow cytometer.

### 4.9. ATP Assays

Intracellular ATP levels in RSC96 cells were measured using an enhanced ATP detection kit (Beyotime Biotechnology, S0027). After lysing RSC96 cells with ATP lysis buffer, the samples were centrifuged at 12,000× *g* for 5 min at 4 °C to collect the supernatant. The supernatant and detection solution were added to a 96-well plate and mixed quickly, and luminescence signals were detected using a TECAN multimode plate reader. The protein concentration in the samples was determined using a BCA protein concentration assay kit (Beyotime Biotechnology, Shanghai, China). ATP concentrations were then expressed as nmol/mg protein.

### 4.10. Western Blot

Total proteins were extracted from cells using a RIPA lysis kit (P0013C, Beyotime Biotechnology, Shanghai, China) and mixed with loading buffer, followed by heating at 95 °C for 30 min. Proteins were separated using 10% or 12% SDS-PAGE and transferred to a 0.45 μM PVDF membrane (IPVH00010, Merck Millipore, Burlington, MA, USA). After blocking with 5% non-fat milk for 1 h, the membrane was incubated overnight at 4 °C. Following three washes with PBS containing 0.1% Tween-20 (T-PBS), the membrane was incubated with secondary antibodies for 1 h. After three additional washes with T-PBS, images were captured using an exposure system (General Electric Company, Boston, MA, USA) and analyzed. The following antibodies were used: anti-GAPDH (db106, 1:10,000, Diagbio, Hangzhou, China), anti-DLAT (T58125, 1:1000, Abmart, Shanghai, China), anti-MPZ (10572-1-AP, 1:1000, Proteintech China (Wuhan Sanying Biotechnology), Wuhan, China), anti-MBP (10458-1-AP, 1:1000, Proteintech), anti-HSP70 (M20033, 1:1000, Abmart), anti-SLC31A1 (T510261, 1:1000, Abmart), anti-FDX1 (12592-1-AP, 1:1000, Proteintech), anti-ATF3 (TD3110S, 1:1000, Abmart) and anti-β-TUBULIN (A12289, 1:1000, Abclonal, Wuhan, China).

### 4.11. Von-Frey Test

Mechanical allodynia was assessed using an electronic von Frey filament test (IITC Life Science Inc., Woodland Hills, CA, USA). C57BL/6J mice were placed on a metal mesh, and the left and right hind paws were stimulated from below using a plastic tip until a clear withdrawal response was observed. The mechanical threshold was automatically recorded by the electronic device.

### 4.12. Gait Analysis

Footprint analysis was conducted four weeks post-drug administration. Different dyes were used to mark the left hind paw (blue) and right hind paw (red) of the mice. Mice were allowed to walk across a blank sheet of paper at a constant speed, and the stride length, stride width, and toe spread distance of both hind paws were measured.

### 4.13. Transmission Electron Microscopy Analysis

Isolated sciatic nerve tissues from C57BL/6J mice were collected with care to avoid mechanical stress. The tissues were fixed in 1 mL of fresh glutaraldehyde (Scientific Phygene, Fuzhou, China, PH9003) at 4 °C for 2–3 days at room temperature. The sciatic nerve tissues were then fixed with 1% osmium tetroxide for 1 h and stained with 2% uranyl acetate for 30 min. After dehydration and embedding, the sciatic nerve tissues were sectioned using an ultrathin slicer. Images were collected using a transmission electron microscope (Talos 120 kV).

### 4.14. Measurement of Intracellular Cu^+^

Intracellular cuprous ion levels were measured using a Cellular Cuprous Fluorometric Assay Kit (E-BC-F102, Elabscience, Wuhan, China). Approximately 1 × 10^6^ cells were resuspended in saline, centrifuged at 10,000× *g* for 10 min, and the supernatant was collected. After a 10 min reaction at 37 °C in the dark, fluorescence values were detected using a fluorescence microplate reader.

### 4.15. Measurement of Intracellular Cu^2+^

Intracellular copper ion levels were assessed using a Copper Colorimetric Assay Kit (E-BC-K775-M, Elabscience). Approximately 1 × 10^6^ cells were lysed on ice for 10 min using the lysis buffer provided in the kit. The samples were centrifuged at 12,000× *g* for 10 min at 4 °C, and the supernatant was incubated with the assay buffer at 37 °C in the dark before measuring absorbance at 580 nm using a microplate reader.

### 4.16. Intracellular Copper Fluorescent Probe Detection

Intracellular cuprous ion levels were detected using a cuprous ion probe (C557, DOJINDO, Kyushu Island, Japan). Approximately 1 × 10^6^ cells were incubated with 5 μM CupprosGreen Working Solution at 37 °C in the dark for 3 h, and fluorescence values were measured using a fluorescence microplate reader (Tecan, Männedorf, Switzerland).

### 4.17. Molecular Docking

Structural data for SLC31A1, HSP70, and FDX1 were downloaded from the PDB database (https://www.rcsb.org/, accessed on 21 November 2025). The molecular structure of EGCG was obtained from PubChem (https://pubchem.ncbi.nlm.nih.gov/, accessed on 21 November 2025). Molecular docking was performed using AutoDock Vina 1.1.2 to visualize the ligand conformations with the lowest binding affinities.

### 4.18. Molecular Dynamics (MD) Simulations

In this study, the protein-ligand complex was constructed using the Amber14SB force field to model the protein, while the ligand was parameterized with the GAFF2 force field incorporating AM1-BCC charges. All molecular dynamics simulations were performed using GROMACS 2024.5. To evaluate the overall conformational stability of the protein during the simulation, RMSD was calculated; additionally, the binding stability of the complex was assessed by monitoring the distance between the geometric centers of the protein and the ligand. These analyses provide systematic and rigorous kinetic evidence for understanding protein-ligand interactions.

### 4.19. GSEA, KEGG, and GO Pathway Enrichment Analysis

To further elucidate the biological functions of the target genes, gene set enrichment analysis (GSEA) software version 4.4.0 was utilized to analyze the protein data from RSC96 cells. Pathways with |NES| > 1, NOM *p*-value < 0.05, and FDR *q*-value < 0.25 were considered significantly enriched. The “ClusterProfiler” package 4.4.4 (R 4.2.0) was employed for KEGG and GO analyses of differentially expressed proteins, with pathways deemed significantly enriched at *p* < 0.05. Visualization of the KEGG and GO results was performed using an online tool (https://cloud.metware.cn/, accessed on 16 October 2025).

### 4.20. Transcription Factor Prediction

We performed systematic prediction and analysis of potential transcription factor binding sites of the SLC31A1 gene using six transcription factor prediction databases: FIMO_JASPAR, PWMEnrich_JASPAR, ENCODE, CHEA, GTRD, and ChIP-Atlas.

### 4.21. Transfection of siRNA Oligonucleotides

Cells were seeded into 6-well plates at a density of 7 × 10^4^ cells per well and cultured until reaching approximately 60% confluence. Transfection of siRNA was performed using JET buffer and JET-PRIME reagent (Polyplus, Strasbourg, France, Cat. No. 101000046) according to the manufacturer’s protocol. siRNA duplexes targeting SLC31A1 or a negative control siRNA were transfected at a final concentration of 20 nM. At 6 h post-transfection, the treatment group was exposed to complete medium containing 12.5 nM BTZ for 48 h, while the control group was maintained in standard culture conditions. Following incubation, cells from both treated and untreated groups were harvested for downstream analyses. The siRNA sequences targeting SLC31A1 and the negative control synthesized by Guannanbio (Hangzhou, China).

SLC31A1 (5′–3′): CGGAUAUAUUUGCUGUGAUUAUAdTdT
Negative Control (5′–3′): UAUAAUCACAGCAAAUAUAUCCGdTdT

### 4.22. Data Analysis

Statistical analyses were conducted using GraphPad Prism 10.1.0 software (GraphPad, San Diego, CA, USA) and ImageJ (version 1.8.0). Comparisons between two groups were performed using *t*-tests, while one-way ANOVA was used for multiple-group comparisons. Data are presented as mean ± standard deviation (SD). A *p*-value of <0.05 was considered statistically significant.

## 5. Conclusions

This study confirms that BTZ upregulates the expression of the transcription factor ATF3, which in turn mediates the increased expression of the copper transporter SLC31A1, causing dysregulation of intracellular copper ion homeostasis and subsequent copper accumulation, and ultimately inducing the development of peripheral neurotoxicity. This overload directly promotes the oligomerization of the mitochondrial protein DLAT and disrupts the iron–sulfur cluster protein FDX1, thereby triggering cuproptosis in Schwann cells and ultimately resulting in peripheral nerve damage. Importantly, (−)-Epigallocatechin Gallate, a bioactive compound from green tea, effectively mitigates BTZ-induced neurotoxicity both in vitro and in vivo by downregulating SLC31A1, restoring copper homeostasis, and inhibiting key processes of cuproptosis.

## Data Availability

The datasets used and/or analyzed during the current study are available from the corresponding author on reasonable request. The mass spectrometry proteomics data have been deposited to the ProteomeXchange Consortium (https://proteomecentral.proteomexchange.org, accessed on 21 January 2026) via the iProX partner repository with the dataset identifier PXD072911.

## Supplementary Materials

The following supporting information can be downloaded at: https://www.mdpi.com/article/10.3390/ijms27083680/s1.

## Abbreviations

ATF3, activating transcription factor 3; BTZ, Bortezomib; EGCG, (−)-Epigallocatechin Gallate; BIPN, Bortezomib-Induced Peripheral Neurotoxicity; MBP, myelin basic protein; MPZ, myelin protein zero; KEGG, Kyoto Encyclopedia of Genes and Genomes; GO, Gene Ontology; GSEA, gene set enrichment analysis; DLAT, Dihydrolipoamide S-acetyltransferase; DEPs, differentially expressed proteins; CQ, Chloroquine; ammonium tetrathiomolybdate (ATTM); ferredoxin 1 (FDX1); Cell Counting Kit-8, CCK-8; transmission electron microscopy, TEM; root mean square deviation, RMSD.
