# Supplementary material for: ATF3/SLC31A1-Mediated Cuproptosis Contributes to Bortezomib-Induced Peripheral Neurotoxicity and Intervention by (−)-Epigallocatechin Gallate
Source: Int J Mol Sci. 2026 Apr 21;27(8):3680. doi: 10.3390/ijms27083680 (PMC13117014; doi:10.3390/ijms27083680)
Supplement: Supplementary file 1 [file ijms-27-03680-s001.zip › ijms-4195171-supplementary.pdf]

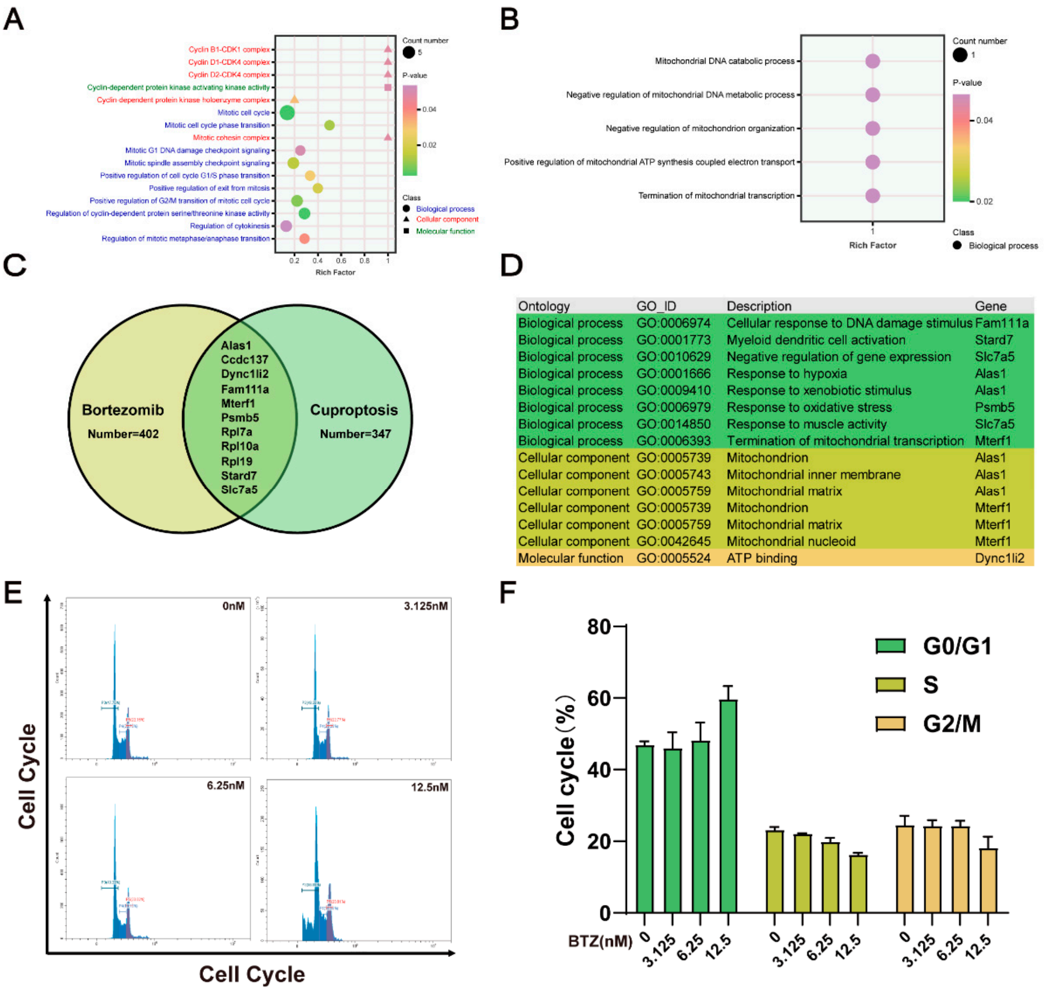

Figure S1. After Rsc96 samples treated by BTZ, Pathway enrichment analysis via GO was performed on cell cycle-related(A) and mitochondrial-related(B)proteins in differentially expressed genes (DEGs). (C)The cross-proteins between the differentially expressed genes (DEGs) in the proteomics of Rsc96 after BTZ treatment and the previously reported copper death-related protein CRG. (D)GO annotation of cross-protein. (E)The cell cycle in Rsc96 treated with BTZ was analyzed by flow cytometry using PI. (F) Graph showing the changes in the cell cycle (n = 3). Data are expressed as mean ± standard deviation (SD).

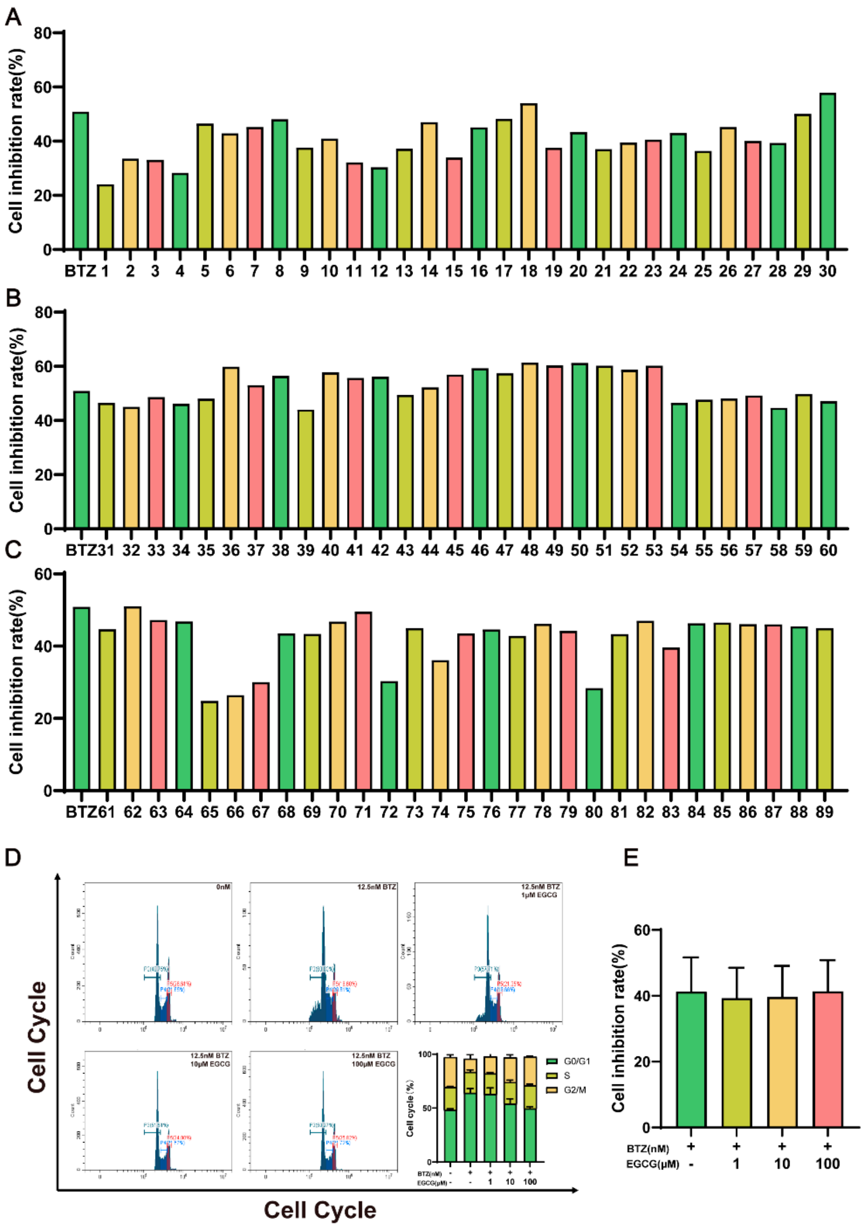

Figure S2. (A-C) After the combined treatment with BTZ and a total of 89 compounds, the cell inhibition rate of Rsc96 for 48h. After the combined treatment with BTZ and EGCG for 48h: (D) the cell cycle in Rsc96 was analyzed by flow cytometry using PI and graph showing the changes in the cell cycle (n = 3). (E) the cell viability of Mino for 48h (n = 3). Data are expressed as mean ± standard deviation (SD).
